# Fusion Partner and ER-Retention Signal Are Associated with Distinct Host Proteomic and Metabolic Responses During Interleukin-15 Production in *Nicotiana benthamiana*

**DOI:** 10.3390/plants15182844

**Published:** 2026-09-17

**Authors:** Alina Savinova, Pipob Suwanchaikasem, Balamurugan Shanmugaraj, Waranyoo Phoolcharoen

**Affiliations:** 1 Center of Excellence in Plant-Produced Pharmaceuticals, Chulalongkorn University, Bangkok 10330, Thailand; 6573006733@student.chula.ac.th; 2 Department of Pharmacognosy and Pharmaceutical Botany, Faculty of Pharmaceutical Sciences, Chulalongkorn University, Bangkok 10330, Thailand; 3 Baiya Phytopharm Co., Ltd., Bangkok 10330, Thailand; pipob.s@baiyaphytopharm.com; 4 Centre for Natural Products and Functional Foods, Karpagam Academy of Higher Education, Coimbatore 641021, India; balasbm17@gmail.com; 5 Department of Biotechnology, Karpagam Academy of Higher Education, Coimbatore 641021, India

**Keywords:** plant molecular farming, recombinant biopharmaceuticals, proteomics, metabolomics, interleukin-15, endoplasmic reticulum retention, recombinant protein production

## Abstract

Plant molecular farming has emerged as a promising platform to produce recombinant biopharmaceuticals. The expression of foreign proteins changes the host proteome and metabolome, and these changes might have an effect on the accumulation of recombinant proteins. In this study, we investigated how fusion partners and ER-retention strategies influence host responses during transient production of recombinant human interleukin-15 (IL-15) in *Nicotiana benthamiana*. Four IL-15 constructs carrying either an 8 × His tag or an IgG1 Fc fusion, with or without an N-terminal signal peptide and C-terminal SEKDEL motif (KD), were compared at 4 days post-infiltration. Western blot analysis detected IL-15 only in the Fc-fusion constructs. Plants expressing IL15-Fc accumulated more antioxidant enzymes and phenylpropanoid-derived phenolics. Expression of IL15-Fc with the SP/KD construct showed increased abundance of proteins involved in protein folding and translation, together with reduced representation of secondary metabolic pathways. Overall, the results demonstrate that fusion partner and SP/KD targeting strategy jointly shape the host response to recombinant IL-15 expression and should therefore be evaluated together during construct design for plant molecular farming.

## 1. Introduction

The production of recombinant therapeutic proteins in plants, commonly referred to as plant molecular farming (PMF), is considered an alternative to microbial and mammalian cell expression systems. Transient expression in *N. benthamiana* has become particularly prominent because of its efficient genetic transformation, reduced risk of contamination with human and animal pathogens, and the capacity of the plants to perform complex post-translational modifications [1,2,3,4,5]. The recent advancements in vector optimization, including modification of the promoters, terminators, and addition of other regulatory elements, have substantially improved transgene expression in plant systems [6,7,8]. To date, vaccine antigens, virus-like particles, monoclonal antibodies, growth factors and peptides have been produced in plants [9,10,11,12,13,14,15,16].

These improvements have increased the range of recombinant proteins that plants can produce, but some still accumulate in lower quantities in plant tissue. Cytokines are one of the most valuable classes of therapeutic proteins because of their role in immune regulation, cell differentiation, and proliferation [17]. Several recombinant cytokines, including IL-2, IL-6, and IL-8, have been produced in plant systems with retained biological activity [18,19]. IL-15 activates natural killer cells and memory CD8+ T cells and is currently under investigation for cancer immunotherapy [20]. However, the production of IL-15 in plant systems is limited due to low accumulation [21]. There are several strategies routinely used to improve protein accumulation: fusion to a stabilizing partner, such as an Fc domain [22], or targeting to specific subcellular compartments, such as the endoplasmic reticulum (ER) [23].

Both strategies increase the load on the host secretory pathway. A fusion partner alters the size, charge and post-translational modification requirements of the product, and this affects how the cell folds and transports it [24]. An ER-retention signal directs the protein into the secretory pathway and retains it in the ER, recruiting chaperones that support folding and assembly [25].

Agroinfiltration and recombinant protein expression may lead to the production of reactive oxygen species (ROS). The superoxide quickly converts into more stable hydrogen peroxide [26]. On entering the cytosol, hydrogen peroxide oxidizes the cysteine residues in kinases, phosphatases and transcription factors, and this leads to activation of the MAPK cascades, to salicylic acid and jasmonate signaling, and to the expression of defense and phenylpropanoid genes [27,28,29,30]. Hydrogen peroxide could also be a marker of the host response, as it leads to the lignification of the cell wall and is reported in agroinfiltrated leaves [31,32]. When the oxidant is not removed, oxidative conditions in the apoplast and the cytosol could lead to protein carbonylation, an irreversible modification that makes the affected protein prone to degradation or to aggregation [33]. Upon recombinant production, the endoplasmic reticulum (ER) also depends on oxidizing conditions, protein disulfide isomerase, and ER oxireductins to form disulfide bonds [34].

Thus, recombinant protein production and the host defense response reshape the plant’s proteome and metabolome, and these changes could affect product yield and quality. Recent studies have applied omics approaches to investigate the responses of *N. benthamiana* to agroinfiltration and heterologous protein expression. Proteomic and metabolomic profiling have revealed that the agroinfiltration process and the subsequent accumulation of recombinant proteins trigger coordinated changes in host defense proteins, ER chaperone machinery, and secondary metabolite pathways [35,36,37]. These studies varied agroinfiltration conditions or one construct feature at a time. Whether the fusion partner and the targeting signal act on the host independently has not yet been tested.

We previously compared two different cytokines carrying the His tag, IL-1 and IL-15, against non-infiltrated and mock-infiltrated controls, and described the host protein quality control and metabolic signatures that accompany agroinfiltration and recombinant protein accumulation in this system [21].

In this study, we compared the transient expression of four IL-15 constructs that differed in their fusion partner (His tag or Fc-fusion) and in the presence or absence of the SP/KD targeting cassette. We hypothesized that the Fc fusion and the SP/KD targeting strategy would induce distinct changes in the host proteome and metabolome and further examined whether their effects were independent. Integrated LC–MS/MS-based proteomics and metabolomics with pathway enrichment analyses were used to identify the pathways differentially affected by the different IL-15 constructs.

## 2. Materials and Methods

### 2.1. Construction of IL15-His and IL15-Fc Expression Cassettes

Human interleukin-15 (IL15) was produced in *N. benthamiana* as two fusion types: His-tagged and Fc-fused. Each version was constructed with and without the SP/KD targeting cassette. All four constructs were assembled from the same three elements. The IL15 moiety is the mature human interleukin-15 polypeptide (UniProt P40933, residues 49–162). In the Fc-fused constructs, IL-15 is linked using a (G_4_S)_3_ linker to the Fc region of human IgG1 (UniProt P01857) comprising a truncated core hinge, CH2 and CH3. In the His-tagged constructs, IL-15 is joined to an 8 × His tag. The constructs carrying the signal peptide and SEKDEL (KD) motif have the murine leader sequence (MGWSCIILFLVATATGVHS) signal peptide at the N-terminus and the SEKDEL at the C-terminus [22]. The sequence of the mature human interleukin-15 was codon-optimized for expression in *N. benthamiana* (GeneArt gene synthesis, Thermo Fisher Scientific). All synthetic genes were cloned into the geminiviral vector pBYR2eK [38] and introduced into *Agrobacterium tumefaciens* strain GV3101 by electroporation.

### 2.2. Transient Expression of Recombinant Proteins in N. benthamiana

*A. tumefaciens* cultures carrying each construct were grown overnight in LB medium (10 g/L tryptone, 5 g/L yeast extract and 10 g/L NaCl) with a mix of selective antibiotics: 50 μg/mL rifampicin, 50 μg/mL gentamicin and 50 μg/mL kanamycin. The bacterial suspension was centrifuged at 6000× *g* for 15 min and resuspended in infiltration buffer (10 mM MES, 10 mM MgSO4, pH 5.5) to a final OD600 of 0.4–0.6. Leaves of 3–4-week-old *N. benthamiana* plants were infiltrated with a 1-mL needleless syringe through the abaxial (stomatal) surface; three to four leaves were infiltrated per plant. After infiltration, the plants were maintained in a controlled environment with a 16 h light and 8 h dark photoperiod at a temperature of 28 °C. The infiltrated leaves were harvested for analysis at 4 days post-infiltration (dpi), a time point that corresponds to the peak accumulation of recombinant protein in this geminiviral vector system and was previously identified as optimal for hIL-15-Fc production in this host in an earlier optimization study conducted by our group [22,38]. The experiments were performed in triplicate.

### 2.3. Protein and Metabolite Extraction

Leaf tissue (around 800–900 mg) was mixed with 200 μL of 50 mM ammonium bicarbonate (ABC) buffer pH 7.5 and disrupted using 3-mm steel balls in a tissue homogenizer SWE-FP Plus (ServiceBio, Wuhan, China) at 70 Hz for 3 rounds for 3 min each, with 15 s pauses, under a controlled temperature of 4 °C. The crude extract was clarified by centrifugation at 14,000× *g* for 10 min at 4 °C. The total protein concentration of the crude extract was determined by Bradford (Bio-Rad, USA) and normalized to 1.3 mg/mL.

For proteomic analysis, the crude extract was reduced with 10 mM dithiothreitol (DTT) at 65 °C for 30 min, alkylated with 500 mM iodoacetamide (IAA) to a final concentration of 25 mM for 20 min in the dark at room temperature and digested overnight at 37 °C with trypsin (at an enzyme-to-substrate ratio of 1:50 (*w*/*w*)). The digestion was quenched by the addition of formic acid to a final concentration of 1.5%. The solution was centrifuged at 14,000× *g* for 10 min at 4 °C. The resulting supernatant was collected and submitted for LC-MS/MS proteomics analysis.

For metabolomic analysis, crude leaf extract was mixed with 100% methanol containing 50 ng/mL sulfadimethoxine as an internal standard and centrifuged at 14,000× *g* for 10 min at 4 °C. The resulting supernatant was collected and submitted for LC-MS/MS metabolomics analysis.

For Western blot analysis, the recombinant proteins were purified from the normalized crude extract using affinity purification. For the His-tagged constructs, 800 μL of crude extract was incubated with 20 μL of Ni Sepharose FF resin (Cytiva, Marlborough, MA, USA) under agitation on ice for 1.5 h. The beads were washed with 50 mM ABC buffer, and the bound protein was eluted with 100 μL of PBS buffer containing 500 mM imidazole. For the Fc-fusion constructs, 800 μL of crude extract was incubated with 20 μL of MabSelect resin (Cytiva, USA) under the same conditions. Following washing, the Fc-fusion protein was eluted with 100 μL of 0.1 M glycine, pH 3.0.

### 2.4. SDS Gel Electrophoresis and Western Blot

Eluted fractions (100 μL) were mixed with 20 μL of 6× reducing loading buffer containing β-mercaptoethanol, and 50 μL of each sample was loaded onto a 15% polyacrylamide gel. SDS-PAGE was carried out at 150 V for 1.5 h using a Mini-PROTEAN Tetra Cell system (Bio-Rad Laboratories, Hercules, CA, USA). The gel was stained with Coomassie Brilliant Blue for total protein visualization. For immunoblotting, proteins were transferred onto an activated PVDF membrane (Bio-Rad, USA). The membrane was blocked with 5% skimmed milk in TBST for 1 h, then incubated overnight at 4 °C with rabbit anti-IL-15 primary antibody (Cat. no. 500-P15, PeproTech, ThermoFisher Scientific, USA) diluted 1:1000. The following day, HRP-conjugated goat anti-rabbit secondary antibody (Cat. no. ab6721, Abcam, Cambridge, UK) diluted 1:2500 was applied and incubated for 1.5 h at room temperature. Protein bands were detected using Amersham ECL Detection Reagent (Cat. no. RPN3031, Cytiva, Marlborough, MA, USA). Images of the Coomassie-stained gel and the Western blot were captured with an iBright Imaging System (ThermoFisher Scientific, Waltham, MA, USA) and analyzed using ImageLab 6.1 software (Bio-Rad, USA).

### 2.5. LC-MS/MS Based Proteomic Profiling

Tryptic-digested peptides were analyzed on an Agilent Peptide Map column (2.1 × 150 mm, 2.7 µm) coupled to an Agilent 6545XT LC-QTOF instrument (Agilent, Santa Clara, CA, USA). Mobile phases consisted of 0.1% formic acid in water (A) and 0.1% formic acid in acetonitrile (B), with a linear gradient from 0 to 90% B applied over 75 min. Full-scan MS1 data were collected across 40–1700 m/z, and precursor ions were selected for data-dependent MS2 fragmentation within 25–1000 m/z [39]. The complete MS parameters are given in Supplementary Table S1. All samples were measured in triplicate.

### 2.6. LC-MS/MS Metabolomic Profiling

The methanolic fraction of crude extract (10 μL injection volume) was separated on the Agilent InfinityLabPoroshell 120 EC-C18 column (2.1 × 100 mm, 2.7 μm). A water/acetonitrile gradient (both containing 0.1% formic acid) running from 0 to 90% over 20 min was used for separation. Mass spectra were acquired in both positive and negative electrospray ionization modes, with MS1 scans spanning 40–1700 m/z and data-dependent MS2 fragmentation covering 25–1000 m/z. The complete MS parameters are given in Supplementary Table S2.

### 2.7. Protein Identification, Statistical and Pathway Analysis

Protein identification was performed using Maxquant 2.8.1.0 software [40]. Raw spectral data were matched against a composite protein sequence database combining the reference proteomes of *N. benthamiana* (Taxonomy ID: 4100), *N. tabacum* (Taxonomy ID: 4097), *N. attenuata* (Taxonomy ID: 49451), *N. sylvestris* (Taxonomy ID: 4096), and *A. tumefaciens* (Taxonomy ID: 176299), obtained from UniProt. The FASTA sequence of IL-15 together with a standard contaminant list was appended to the database. Precursor mass tolerance was initially set at 20 × 10^−6^ (20 ppm, parts per million) and narrowed to 10 × 10^−6^ (10 ppm, parts per million) during the main search. Label-free quantification relied on the MaxLFQ algorithm [41]. A target-decoy strategy was applied at both the peptide-spectrum match and protein match, maintaining a 1% false discovery rate (FDR).

Downstream quantitative analysis was carried out in Perseus (v1.6.14.0) software [42]. Entries flagged as reverse hits, contaminants, or identified solely through modified peptides were excluded. LFQ intensity values underwent log2 transformation and were filtered so that each retained protein had valid measurements in at least 70% of samples. Remaining missing values were replaced by random draws from a down-shifted normal distribution (width 0.3 SD, shift 1.8 SD). Data quality was assessed through principal component analysis, Pearson correlation, and intensity distribution plots. Hierarchical clustering was performed using Euclidean distances with Ward’s minimum-variance linkage applied to Z-scored values. Statistically significant abundance changes were determined by a two-tailed Student’s *t*-test corrected for multiple testing with the Benjamini–Hochberg procedure (FDR < 0.05, S0 = 0.1). Differentially abundant proteins were annotated with KEGG Orthology (KO) identifiers, and the resulting KO set was used as the query for pathway enrichment analysis. The KEGG pathway maps of *Arabidopsis thaliana (ath)*, *Nicotiana tabacum (nta)*, *Nicotiana attenuata (nau)* and *Nicotiana sylvestris (nsy)* were used as the background for the enrichment analysis. Enrichment was calculated with Fisher’s exact test, and *p*-values were corrected with the Benjamini–Hochberg procedure across both pairwise comparisons and both directions of change; pathways were considered significantly enriched at FDR < 0.05.

### 2.8. Metabolite Identification, Statistical and Pathway Analysis

Raw metabolomic data were processed in MS-DIAL 5.5 software [43], which handled peak picking, spectral deconvolution, chromatographic alignment, and compound annotation. Metabolite identities were assigned by matching experimental MS/MS spectra to the RIKEN ReSpect phytochemical library [44], the Fiehn/Vaniya Natural Products Library [45], the Global Natural Products Library [46] and a validated ESI(+/−)-MS/MS reference collection (version 17). Signal intensities were normalized with the LOWESS algorithm and the internal standard. Features passing all the following filters were retained: dilution–QC Pearson correlation coefficient ≥ 0.70, pooled-QC coefficient of variation ≤ 30%, mass accuracy within ±20 × 10^−6^ (20 ppm), and composite identification score ≥ 0.7.

Curated feature tables were then imported into MetaboAnalyst 6.0 software [47] for multivariate and univariate analyses. Heatmaps were constructed after log2 transformation and hierarchical clustering with Euclidean distances and Ward’s minimum-variance linkage applied to Z-scored values. Differentially abundant metabolites were identified using pairwise Student’s *t*-test with the Benjamini–Hochberg correction for multiple testing (FDR < 0.05), and with an effect-size cutoff of |log2 FC| ≥ 1 (i.e., ≥2-fold change). Significantly different metabolites were annotated with KEGG compound identifiers. The resulting compounds dataset was used as a query in the impact pathway plot analysis. Because downstream pathway mapping and enrichment analysis require compounds to be linked to metabolic networks, only significant features with a valid KEGG identifier were retained for pathway-level interpretation. Features lacking a KEGG annotation were reported but excluded from enrichment analysis. To define the background for the impact plot, KEGG pathway maps of *Arabidopsis thaliana (ath)*, *Nicotiana tabacum (nta)*, *Nicotiana attenuata (nau)* and *Nicotiana sylvestris (nsy)* were used. Enrichment was calculated with Fisher’s exact test and corrected as described for the proteomic data.

## 3. Results and Discussion

### 3.1. Transient Expression of IL15-His and IL15-Fc Constructs in N. benthamiana

**Figure 1:**
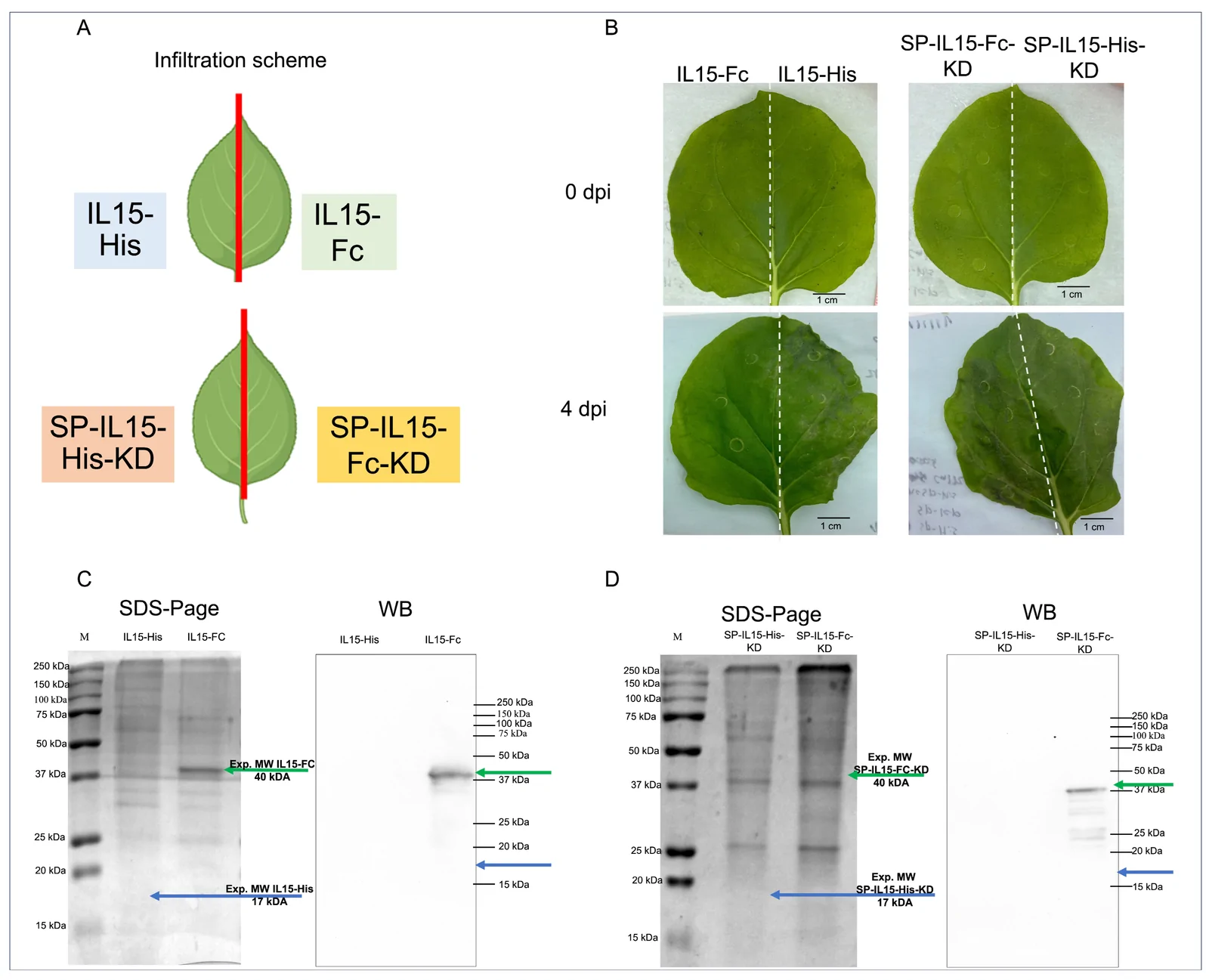
Transient expression of four IL-15 constructs in *N. benthamiana*. (**A**) Infiltration scheme. Constructs differing only in the fusion partner were infiltrated into opposite halves of the same leaf. (**B**) Representative infiltrated leaves at 0 (top) and 4 (bottom) days post-infiltration (dpi). Dashed lines mark the boundary between the two infiltrated halves. (**C**,**D**) SDS-PAGE and Western blot (WB) of affinity-purified crude extract for IL15-His and IL15-Fc (**C**) and for SP-IL15-His-KD and SP-IL15-Fc-KD (**D**): His-tagged constructs on Ni-NTA resin and Fc-fused constructs on Protein A resin. M, molecular weight marker; green arrows, expected mass of the Fc-fused protein (40 kDa); blue arrows, expected mass of the His-tagged protein (17 kDa).

Leaves of 3–4-week-old *N. benthamiana* were infiltrated with *A. tumefaciens*, carrying each of the four IL-15 constructs. Each pair, differing only in the fusion partner, was infiltrated into opposite halves of the same leaf. IL15-His and IL15-Fc could be compared within a single leaf, without leaf-to-leaf variation (Figure 1A). By 4 dpi, infiltrated tissues exhibited mottling (irregular pale patches), marginal curling, and localized necrosis. These symptoms were more pronounced in leaves expressing the constructs carrying the SP/KD targeting cassette (Figure 1B). All four constructs were delivered using the same *A. tumefaciens* strain and optical density, and were harvested at the same time point. Thus the observed differences in tissue appearance were associated with construct type under the experimental conditions used. The extent of tissue damage was assessed visually and was not quantified.

Protein accumulation was assessed by SDS-PAGE and Western blot analysis (Figure 1C,D). Without the SP/KD targeting cassette, IL15-Fc gave a clear band at approximately 37–40 kDa, while IL15-His gave no detectable signal. The pair carrying the signal peptide and KD motif behaved the same way: SP-IL15-Fc-KD produced a band at approximately 37–40 kDa, and SP-IL15-His-KD remained undetectable (Figure 1D, Supplementary Figures S1 and S2). The absence of a band indicates accumulation below the detection limit of the assay rather than the absence of expression, so a more sensitive method was needed before any comparison between the constructs could be interpreted.

Tryptic digests of unpurified leaf tissue from all four constructs were analyzed by LC-MS/MS peptide mapping. The tryptic peptide DVQLLENWVNVISDLKK spans the N-terminus of all four constructs, comprising six vector-derived residues followed by the first eleven residues of mature IL-15, and has no significant match in the *N. benthamiana* proteome. It was extracted at its accurate mass in two charge states (m/z 1007.0544 for the doubly charged and 671.7053 for the triply charged form) and gave a single peak between 41.73 and 41.84 min in all twelve tissue samples, with no corresponding peak in either injection blank (Supplementary Figure S4). Its identity was confirmed by MS/MS fragmentation in the Fc-fused samples only, at a mass error of −3.3 ppm (Supplementary Figure S3). IL-15 was present in tissue expressing the His-tagged constructs, but at a level too low for detection by either Western blot or MS/MS identification.

In this study, the fusion partner rather than the SP/KD targeting cassette was associated with whether IL-15 accumulated to detectable levels. Adding the SP/KD targeting cassette did not rescue the His-tagged constructs and removing it did not prevent the Fc-fused ones from accumulating. This looks different from what the processing requirements of the Fc-fusion partner and the His tag would predict. The Fc-fusion partner should fold into the disulfide-linked homodimer and is normally N-glycosylated, so it depends on the folding capacity and glycosylation machinery of the host. A poly-histidine fusion partner added fewer than ten residues to the construct and needs no folding, disulfide bond formation or glycosylation. The more demanding partner nevertheless gave a higher accumulation.

The poly-histidine tag is not the limitation itself. In our previous study, His-tagged IL-1 accumulated to a detectable level, whereas the His-tagged IL-15 remained undetectable [21]. The constraint is specific to IL-15, and one possible explanation is the enhanced stability of the Fc fusion. A globular Fc domain is also a poorer substrate for host proteases than a small unstructured cytokine, so that greater proteolytic stability may contribute to the difference between the two fusion partners. Comparing IL15-Fc with IL15-His within the identical subcellular localization strategy addresses the question that is relevant to production: how the host responds to one construct design rather than the other, when the cytokine and its subcellular destination are held constant. If the fusion partner acted only through the amount of protein accumulated, its effect on the host would not depend on subcellular localization. Any difference between the two pairs indicates that the two features act together. To examine these responses at the molecular level, we next analyzed the proteome and metabolome of leaf tissue expressing each of the four constructs.

### 3.2. The Four IL-15 Constructs Gave Distinct Proteomic and Metabolomic Profiles in N. benthamiana

To capture the host molecular responses associated with each construct, both proteomic and metabolomic profiles were collected from the same leaf tissue samples at 4 dpi. Both datasets come from the same tissue, so the protein and metabolite responses share one biological background. After filtering of low-confidence features, the proteomic dataset comprised 107 proteins and the metabolomic dataset comprised 863 features in positive ion mode and 429 in negative ion mode (Supplementary Tables S3 and S4).

**Figure 2:**
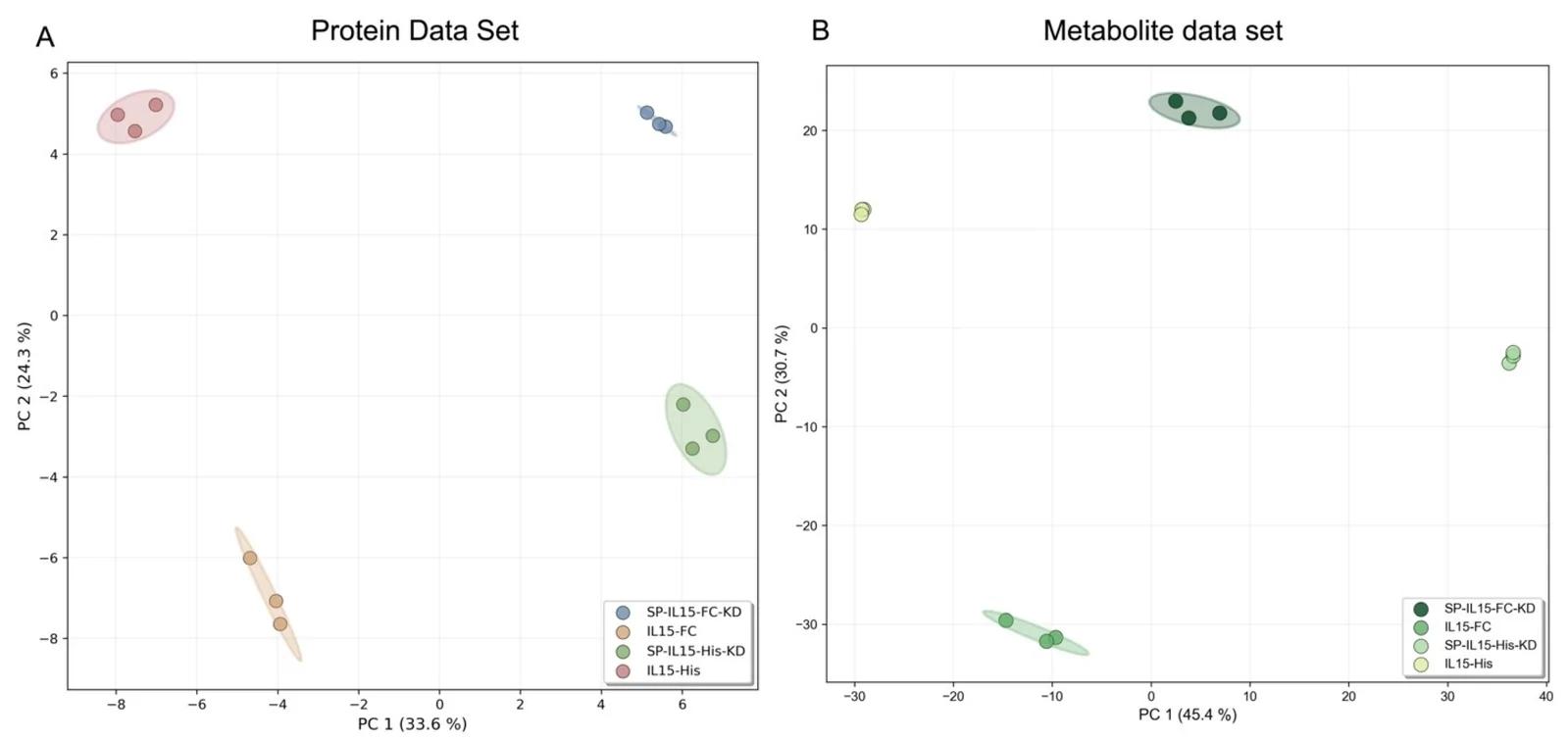
Principal component analysis (PCA) of the (**A**) log2-transformed LFQ intensities for proteomic data set and (**B**) log2-transformed normalized intensity for metabolomic dataset across four different IL15 constructs. (IL15-Fc, IL15-His, SP-IL15-Fc-KD and SP-IL15-His-KD). PC1 on the X-axis and PC2 on the Y-axis. Each dot in the plots represents an individual biological replicate of each group. Different colors refer to different groups. Axis labels give the principal component number, and the percentage in parentheses gives the proportion of total variance explained by that component. Scores are expressed in log_2_ intensity units. Ellipses denote 95% confidence regions assuming a multivariate normal distribution.

Principal component analysis (PCA) was used to provide an unsupervised approach to visualize overall relationships among the four construct groups in the proteomic and metabolomic datasets. In the proteomic dataset, each construct was associated with a distinguishable profile (Figure 2A). The metabolomic data showed the similar pattern: the four construct groups showed clear separation and relatively close clustering of biological replicates (Figure 2B). These results indicate that the different IL-15 constructs were associated with distinct global proteomic and metabolomic profiles.

**Figure 3:**
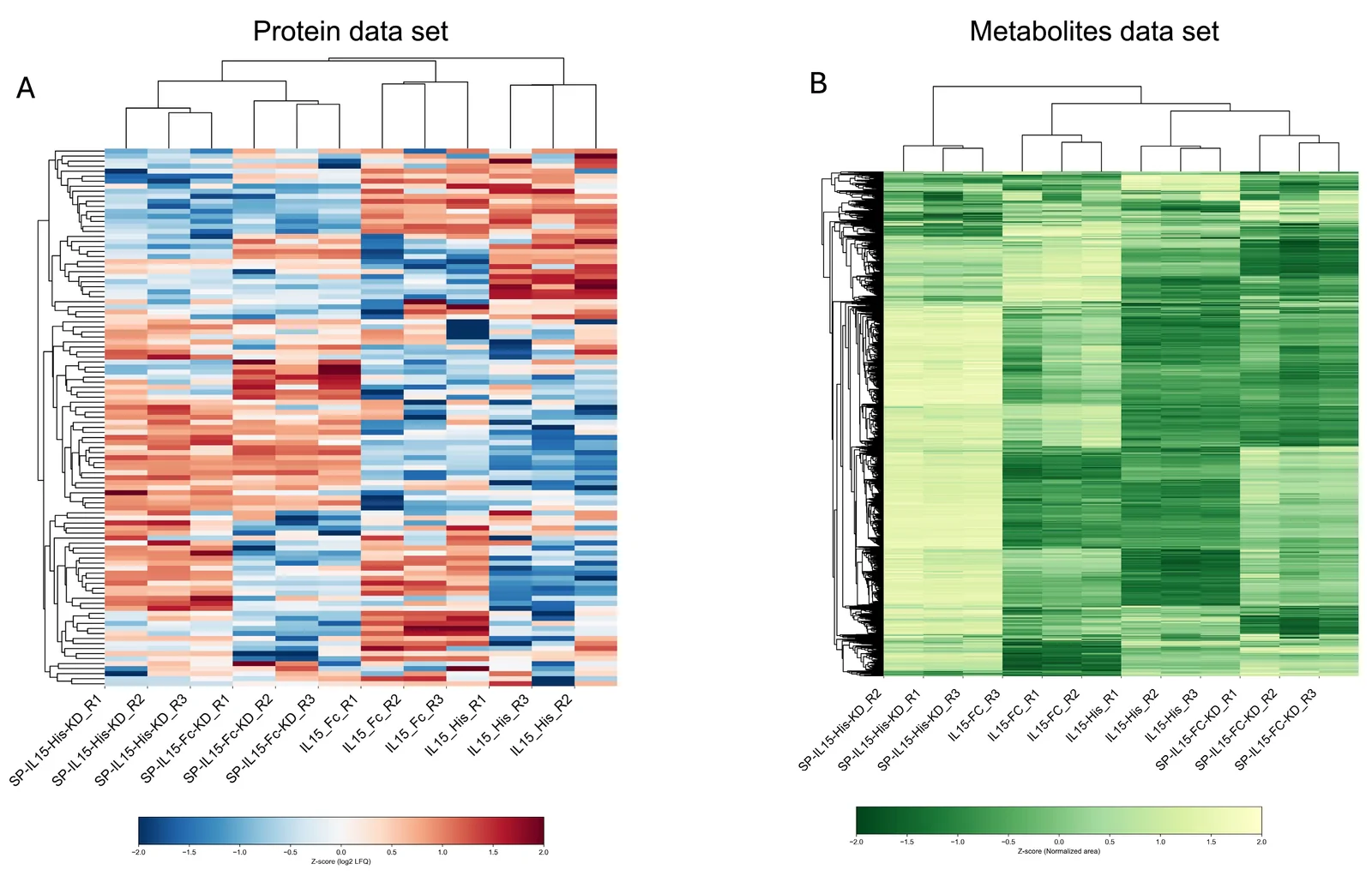
Hierarchical clustering heatmaps of the proteomic (**A**) and metabolomic (**B**) datasets. Columns represent individual samples; rows represent identified proteins (**A**) and metabolites (**B**). Rows and columns were clustered independently by hierarchical clustering with Ward’s minimum-variance linkage on Euclidean distances computed from log_2_-transformed intensities that were Z-scored across samples within each row. The colour scale encodes the row-wise Z-score: in (**A**) proteomic dataset: red indicates high and blue low relative abundance; in (**B**) metabolomic dataset: lime green indicates high and dark green low relative abundance.

Hierarchical clustering (Figure 3) supported the patterns observed by PCA. In the protein dataset (Figure 3A), the samples were clustered primarily by the presence or absence of the SP/KD targeting cassette and then by fusion partner (Fc or His). The IL15-His group formed a separate branch from the other three groups, and the IL15-Fc group was also separated from the SP-IL15-His-KD and SP-IL15-Fc-KD groups. In the metabolite dataset (Figure 3B), the clustering differed from the protein heatmap: the SP-IL15-His-KD group formed a separate cluster from the other three groups, and IL15-His clustered together with SP-IL15-Fc-KD.

### 3.3. The Host Response Depends on the Combination of the SP/KD Targeting Cassette and Fusion Partner

**Figure 4:**
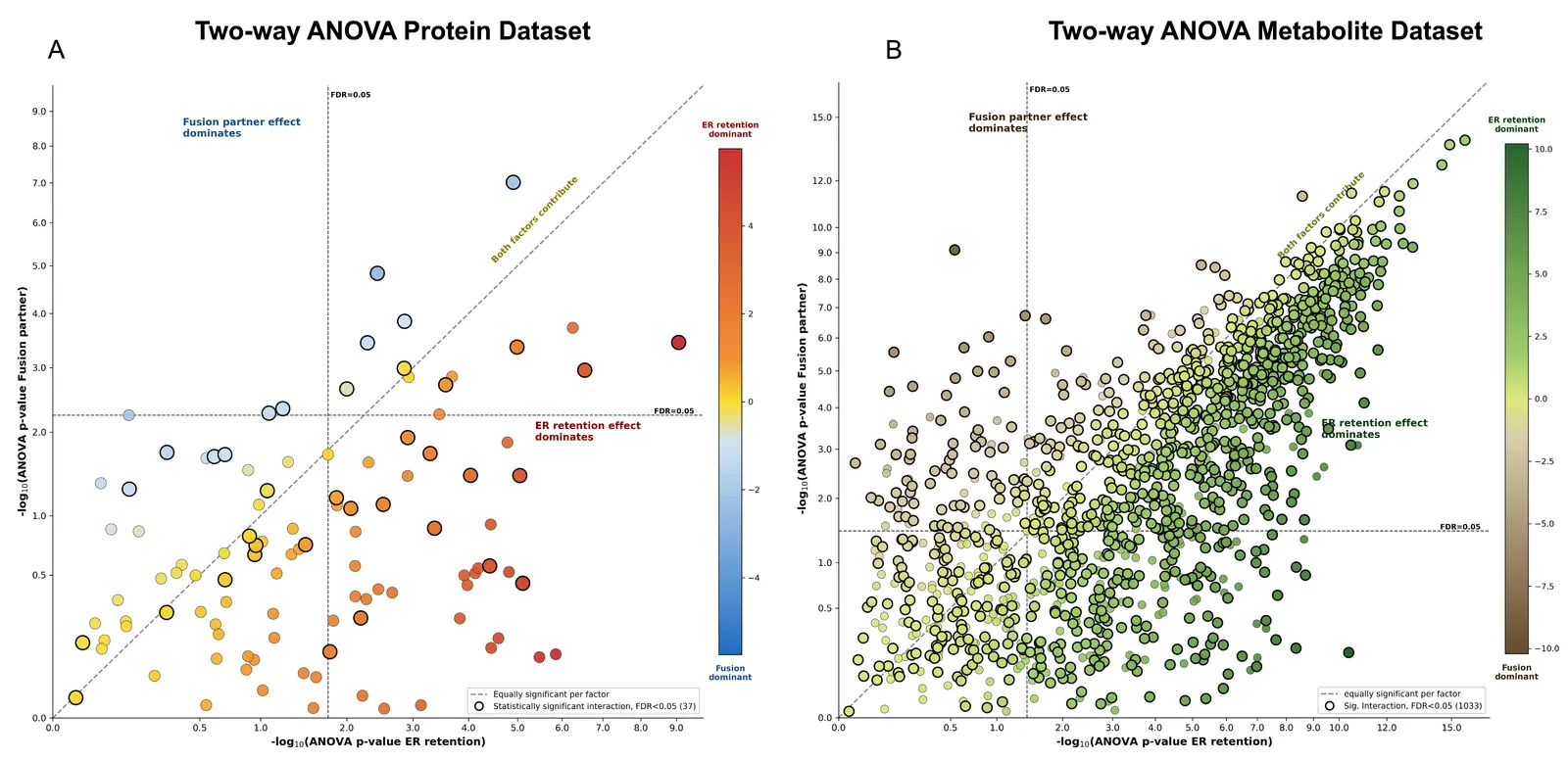
Two-way ANOVA of fusion partner and SP/KD targeting cassette effects on the host proteome and metabolome. Each point is one protein (**A**) or one metabolite feature (**B**). The x-axis shows −log_10_ of the raw *p*-value for the SP/KD targeting cassette main effect, and the y-axis shows −log_10_ of the raw *p*-value for the fusion-partner main effect. Dashed lines mark the −log_10_ *p*-value corresponding to a Benjamini–Hochberg FDR of 0.05, corrected separately for each factor; points beyond a dashed line are significant for that factor. The diagonal marks equal significance for both factors. Color indicates which effect dominates: red in (**A**) and green in (**B**) for SP/KD-cassette-dominant features, blue in (**A**) and brown in (**B**) for fusion-partner-dominant features, and pale yellow for features where the two effects are comparable. Black outlines mark features with a significant interaction term.

A two-way ANOVA was applied to both datasets to separate the two factors and test whether they act independently. Multiple testing was controlled with the Benjamini–Hochberg procedure at an FDR threshold of 0.05 (Supplementary Table S5). Results are presented as a factor-by-factor significance plot in which dot colour indicates which main effect reached significance and features with a significant interaction term are outlined in black (Figure 4).

In the proteomic dataset, 52 proteins responded significantly to the SP/KD targeting cassette factor, 17 to the fusion partner, and 37 to the interaction (Figure 4A, Supplementary Table S5). SP/KD targeting cassette influenced about three times as many proteins as the fusion partner and was the stronger of the two main effects at the protein level. The 37 interaction-significant proteins suggest that the host plant did not respond to each factor in isolation, but to the combination of both factors.

In contrast to the proteomic analysis, where the SP/KD targeting cassette dominated, the metabolome showed near-equal sensitivity to both main effects: 992 features across positive and negative mode responded to the SP/KD targeting cassette and 810 to the fusion partner. The interaction term, however, was significant for 1033 features, more than either main effect alone, indicating that the two factors are more interdependent at the metabolite level than at the protein level. Because each term was tested separately for every feature, these counts are overlapping sets, and their sum exceeds the number of features quantified.

The categories overlap because each feature was tested against both main effects and the interaction separately. The SP/KD targeting cassette affected at least as many proteome and metabolome features as the fusion partner. The two factors also interacted, so the effect of the fusion partner depended on whether the SP/KD targeting cassette was present. This is the reverse of the pattern seen on the Western blot, where detection of the recombinant protein was independent of the SP/KD targeting cassette. Constructs that are indistinguishable by Western blot nevertheless elicited different host responses, and the peptide-level signal in the Fc-fused construct exceeded that in its His-tagged counterpart in both pairs, so a difference that keeps the same sign in the two pairs cannot by itself account for opposite host responses. The two-way ANOVA identified which factors were associated with the host response, but it could not show the direction of change. To establish which proteins and metabolites were upregulated and downregulated, the differential expression analysis was performed.

### 3.4. Differential Protein Abundance and Pathway Enrichment Reveal the Effect of the Fusion Partner on the Leaf Proteome Under Two Subcellular Targeting Strategies

**Figure 5:**
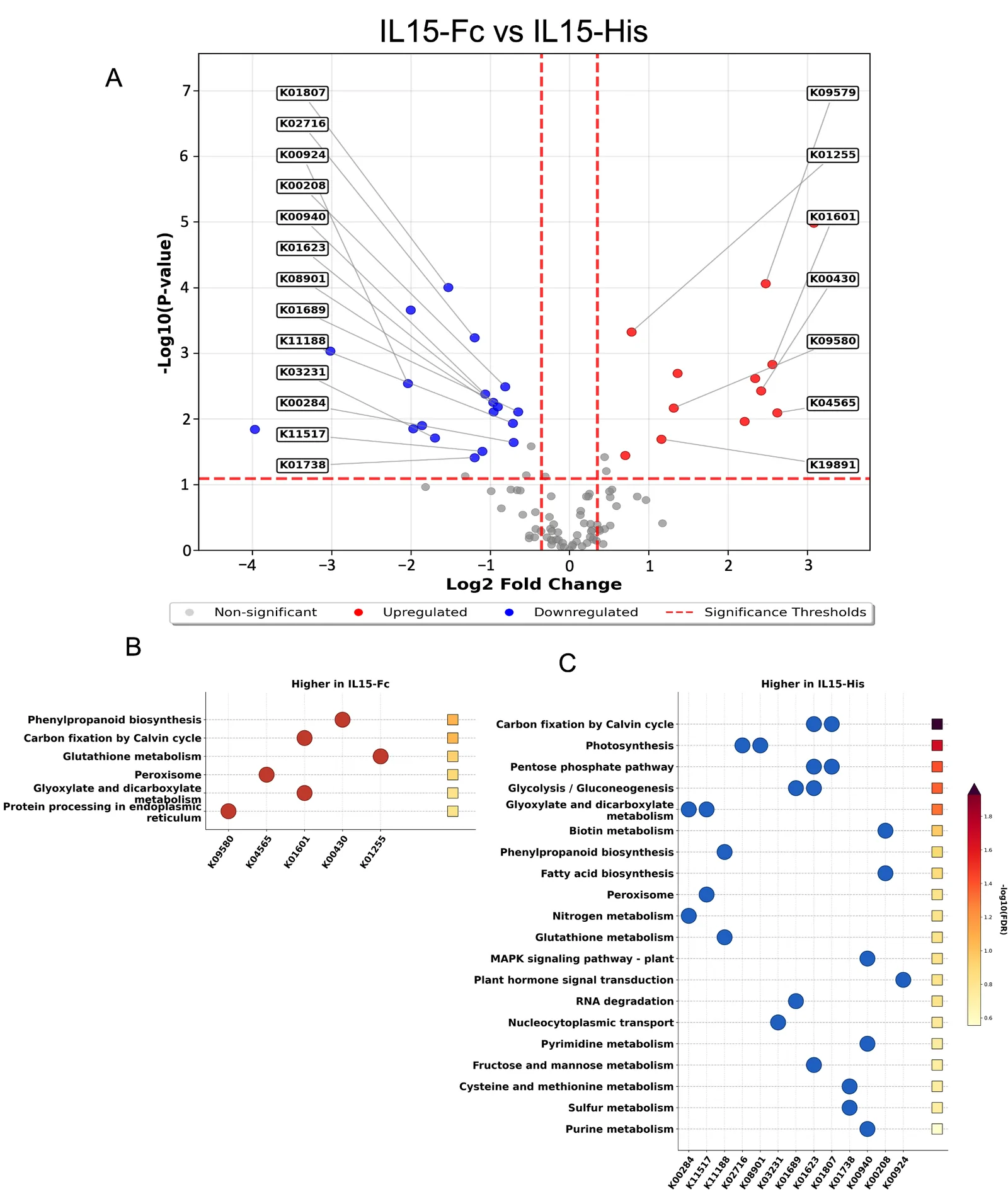
Differential protein abundance and pathway enrichment for IL15-Fc versus IL15-His. (**A**) Volcano plot of differentially expressed proteins between the IL15-Fc versus IL15-His constructs. Red and blue points mark proteins significantly higher in the Fc-fused construct and in its His-tagged counterpart, respectively; the x-axis shows log_2_ fold change and the y-axis shows −log_10_ of the *p*-value; vertical dashed lines mark |log_2_ fold change| = 1 and the horizontal dashed line marks the Benjamini–Hochberg FDR threshold of 0.05 with S0 = 0.1, and only significant proteins carrying a KEGG Orthology identifier are labelled. (**B**,**C**) KEGG pathway enrichment of the significant proteins from the IL15-Fc versus IL15-His comparison, separated by direction: pathways enriched among proteins higher in IL15-Fc (**B**) and higher in IL15-His (**C**). Each row is a pathway, filled circles mark the KEGG identifiers assigned to it, and the colour strip encodes pathway significance as the Benjamini–Hochberg-corrected *p*-value; pathways were considered significantly enriched at FDR < 0.05.

Pairwise differential expression analysis was first carried out for the constructs without the SP/KD targeting cassette, IL15-Fc versus IL15-His (Figure 5A–C, Supplementary Table S6). The comparison was made in the direction of the Fc-fused construct against its His-tagged counterpart, so a positive fold change indicates higher abundance in IL15-Fc. In total, 31 proteins reached significance, 12 of them higher in IL15-Fc and 19 higher in IL15-His. Of these, 21 carried a KEGG Orthology (KO) identifier and were used for pathway enrichment (Figure 5B,C, Supplementary Table S8). Enrichment significance was assessed after Benjamini–Hochberg correction, pooled across both comparisons and both directions, and pathways were called enriched only at FDR < 0.05.

Agroinfiltration and transient expression of a recombinant protein activate oxidative stress responses in *N. benthamiana*, and all four groups in this study carried the same background [21,31]. The comparisons below describe how the constructs differed from this common stressed state. In the IL15-Fc versus IL15-His pair, the antioxidant proteins fell on opposite sides of the comparison. Peroxidase (K00430) and superoxide dismutase [Cu-Zn] (K04565) were among the most strongly upregulated proteins in IL15-Fc, while a glutaredoxin-dependent peroxiredoxin (K11188) and peroxiredoxin Q were higher in IL15-His (Figure 5A, Table 1). These proteins participate in different branches of the peroxide reduction. Superoxide dismutase converts superoxide to hydrogen peroxide, and peroxidase then reduces that hydrogen peroxide to water, using phenolic compounds as electron donors, so that the two enzymes act sequentially [32,48]. The peroxiredoxin route runs in the opposite direction. A peroxiredoxin reduces hydrogen peroxide at its catalytic cysteine and must then be re-reduced by thioredoxin or glutaredoxin. Glutaredoxin, in turn, takes electrons from glutathione, which becomes oxidized in the process [49,50,51]. Mechanistically, this explains why these proteins map to phenylpropanoid biosynthesis, glutathione metabolism and the peroxisome pathway (Figure 5B,C). However, none of these pathways were significantly enriched after correction, so the antioxidant response is best read at the level of the individual enzymes, and this interpretation remains exploratory. Reactive oxygen species, antioxidant enzyme activity and markers of oxidative damage were not measured in this study, and direct measurement of them would be needed before the changes in enzyme abundance could be linked to a defined physiological state.

The abundance of primary metabolism proteins also changed. The RuBisCO large chain (K01601) was higher in IL15-Fc, whereas ribose-5-phosphate isomerase (K01807), fructose-bisphosphate aldolase (K01623), enolase (K01689) and the oxygen-evolving proteins of photosystem II (K02716, K08901) were all higher in IL15-His. These proteins map to carbon fixation by the Calvin cycle, central sugar metabolism (the pentose phosphate pathway and glycolysis), photosynthesis and the glyoxylate and dicarboxylate pathway, all of which were significantly enriched among the proteins higher in IL15-His (Figure 5C, Table 1).

Folding machinery proteins were also more abundant in IL15-Fc. Protein disulfide-isomerase (K09580) and peptidyl-prolyl cis-trans isomerase (K09579) were higher in IL15-Fc, and they both annotate to the protein processing in the endoplasmic reticulum pathway (Figure 5B). This pathway was not significantly enriched, but the higher abundance of this class of proteins is consistent with the folding and disulfide-bond requirements of the Fc partner, which do not apply to the short His-tagged construct.

**Figure 6:**
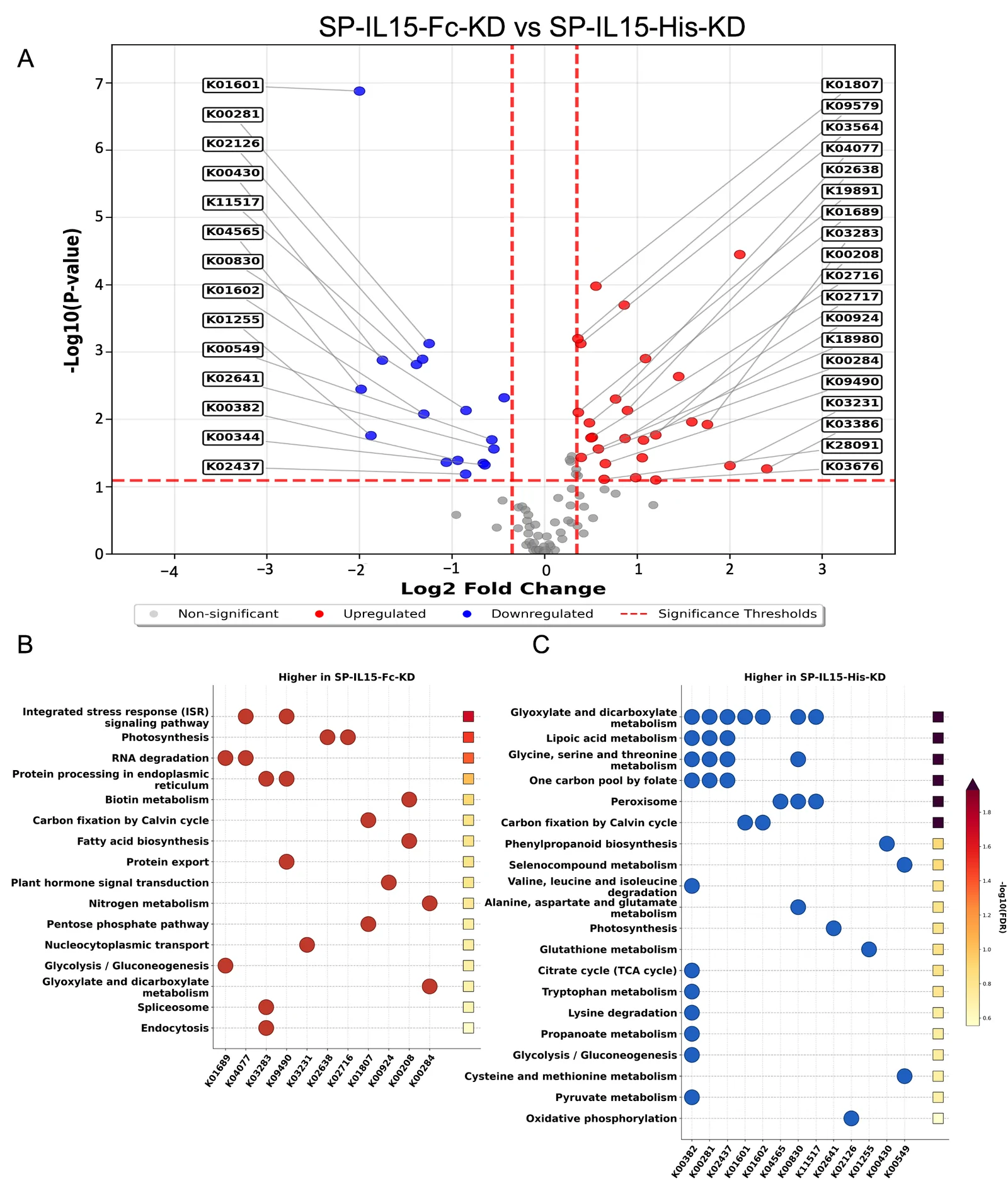
Differential protein abundance and pathway enrichment for the SP-IL15-Fc-KD versus SP-IL15-His-KD. (**A**) Volcano plot of differentially expressed proteins between the SP-IL15-Fc-KD and SP-IL15-His-KD constructs. Red and blue points mark proteins significantly higher in the Fc-fused construct and in its His-tagged counterpart, respectively; the x-axis shows log_2_ fold change and the y-axis shows −log_10_ of the *p*-value; vertical dashed lines mark |log_2_ fold change| = 1 and the horizontal dashed line marks the Benjamini–Hochberg FDR threshold of 0.05 with S0 = 0.1, and only significant proteins carrying a KEGG Orthology identifier are labelled. (**B**,**C**) KEGG pathway enrichment of the significant proteins from SP-IL15-Fc-KD versus SP-IL15-His-KD: pathways enriched among proteins higher in SP-IL15-Fc-KD (**B**) and higher in SP-IL15-His-KD (**C**). Each row is a pathway, filled circles mark the KEGG identifiers assigned to it, and the colour strip encodes pathway significance as the Benjamini–Hochberg-corrected *p*-value; pathways were considered significantly enriched at FDR < 0.05.

To test whether this response depended on the subcellular localization, the same analysis was applied to the SP-IL15-Fc-KD versus SP-IL15-His-KD pair (Figure 6A–C, Supplementary Table S6). In total, 44 proteins reached significance, 27 of them higher in SP-IL15-Fc-KD and 17 higher in SP-IL15-His-KD. Of these, 33 entries carried a KO identifier (Figure 6A, Supplementary Table S6). Setting the two comparisons side by side shows that the direction of the fusion-partner effect changed with the targeting strategy for several of the same proteins.

The antioxidant enzymes were reversed. Peroxidase (K00430) and superoxide dismutase (K04565), which were higher in IL15-Fc, were now higher in the His construct in the presence of the SP/KD targeting cassette. The thiol antioxidants were upregulated as well: two thioredoxin-dependent peroxiredoxins (K03564, K03386), a glutaredoxin (K03676) and peroxiredoxin Q were higher in SP-IL15-Fc-KD. RuBisCO (K01601) reversed as well and was the most strongly downregulated protein in SP-IL15-Fc-KD (Figure 6A, Table 1). Oxygen-evolving enhancer protein 1 of photosystem II (K02716) also reversed and was higher in SP-IL15-Fc-KD, together with the 23 kDa subunit of the oxygen-evolving system. The pathway level followed the same reverse pattern. Carbon fixation by the Calvin cycle remained significant among the proteins higher in the His-tagged construct carrying the SP/KD targeting cassette, whereas the pentose phosphate pathway, glycolysis and photosynthesis were no longer significant (Figure 6C). The glyoxylate and dicarboxylate pathway, glycine, serine and threonine metabolism, the one-carbon pool by folate, lipoic acid metabolism and the peroxisome pathway took their place and became significant among the proteins higher in SP-IL15-His-KD.

There were also features specific to the presence of the SP/KD targeting cassette. A set of folding proteins rose only in SP-IL15-Fc-KD: chaperonin 60 subunit beta 2 (K04077), luminal-binding protein 5 (K09490) and heat shock cognate 70 kDa protein 2-like (K03283) (Figure 6A, Table 1). The integrated stress response signaling pathway, which these proteins represent, was enriched among the proteins higher in SP-IL15-Fc-KD (Figure 6B). Their coordinated increase is consistent with an unfolded protein response under the higher ER protein load imposed by SP/KD-mediated ER targeting of the Fc-fusion [52].

The unfolded protein response increases the amount of chaperone in the endoplasmic reticulum, and it also increases the machinery that returns misfolded proteins to the cytosol for ubiquitination and degradation by the proteasome, so ER-associated degradation is part of the same response [53,54]. A higher chaperone level is therefore compatible with more folding support and with a larger share of the protein being removed, and abundance alone does not separate the two. The degradative side is not covered by the present data. Ubiquitin was identified in both comparisons (Supplementary Table S3) but did not differ significantly in either of them (Supplementary Table S6), and no proteasome subunit or component of the retrotranslocation machinery was identified. Ubiquitin abundance would be a weak indicator in any case, because ubiquitin and the proteasome are recycled after each round of degradation, so neither has to become more abundant when more protein is removed [55]. The contribution of ER-associated degradation and of cytosolic protein quality control to the responses described here therefore remains open.

Two comparisons show that the fusion partner and the subcellular targeting strategy do not act independently at the protein level. The SP/KD targeting cassette did not change whether the recombinant protein was detected by Western blot at 4 dpi, yet it reversed the direction of the antioxidant enzymes, the peroxiredoxins and RuBisCO, and it added a photorespiratory signature and a chaperone response that were absent without retention. Several of these pathways are defined by their metabolites as much as by their enzymes, so the metabolome was analyzed next.

### 3.5. Differential Metabolite Abundance and Pathway Enrichment Are Consistent with the Direction of the Proteomic Response and Its Reversal in the Presence of the SP/KD Targeting Cassette

**Figure 7:**
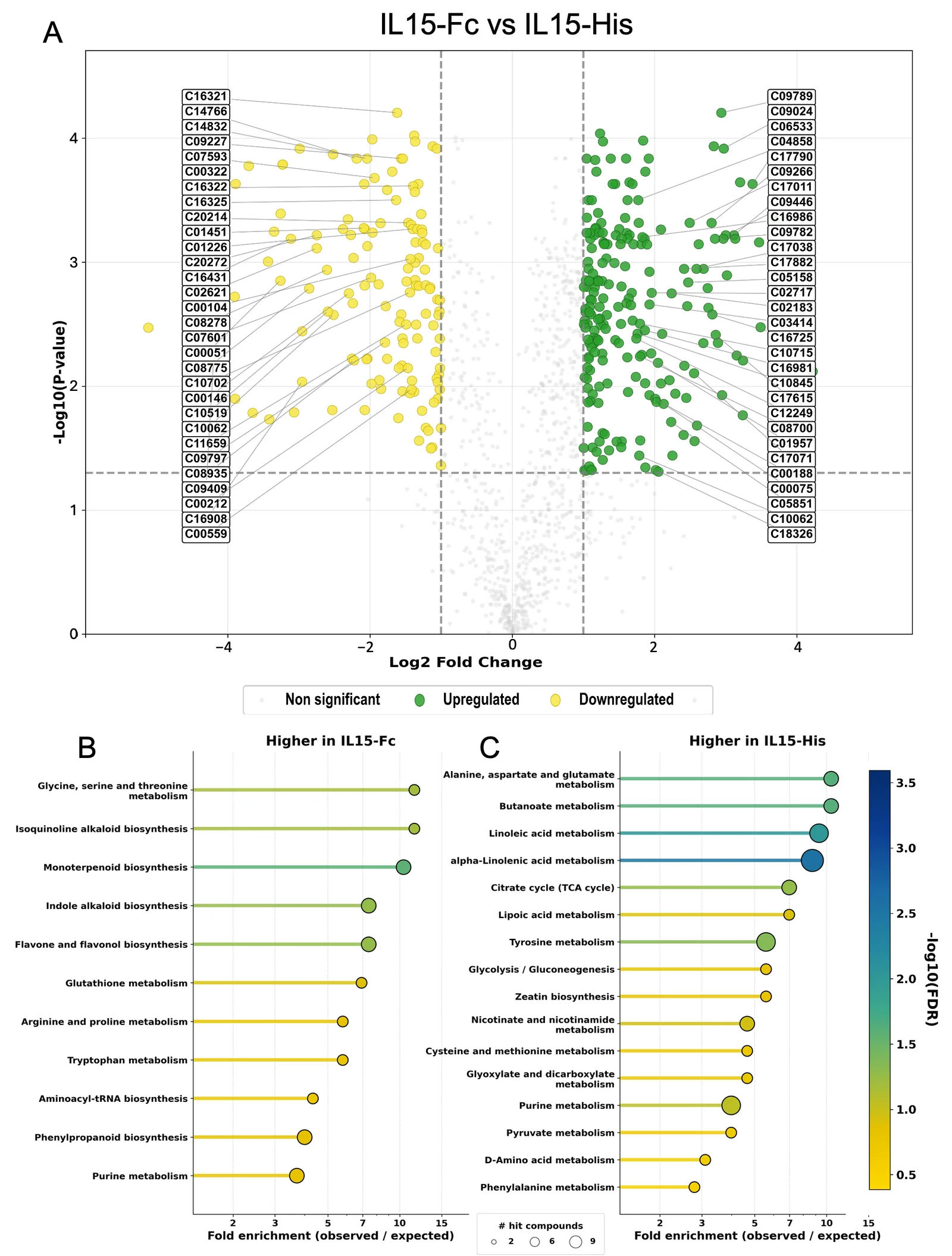
Differential metabolite abundance and pathway enrichment for IL15-Fc versus IL15-His. (**A**) Volcano plot of differentially abundant metabolites between the IL15-Fc and IL15-His constructs. Green and yellow points mark metabolites significantly higher in the Fc-fused construct and in its His-tagged counterpart, respectively; the x-axis shows log_2_ fold change and the y-axis shows −log_10_ of the *p* value; vertical dashed lines mark |log_2_ fold change| = 1 and the horizontal dashed line marks the Benjamini–Hochberg FDR threshold of 0.05, and only significant features carrying a KEGG compound identifier are labelled. (**B**,**C**) KEGG pathway enrichment of the significant metabolites from the IL15-Fc versus IL15-His comparison, separated by direction: pathways enriched among metabolites higher in IL15-Fc (**B**) and higher in IL15-His (**C**). The x-axis gives the fold enrichment of each pathway (observed over expected), bubble size gives the number of hit compounds, and color encodes the Benjamini–Hochberg-corrected *p*-value as −log_10_(FDR); pathways were considered significantly enriched at FDR < 0.05.

**Figure 8:**
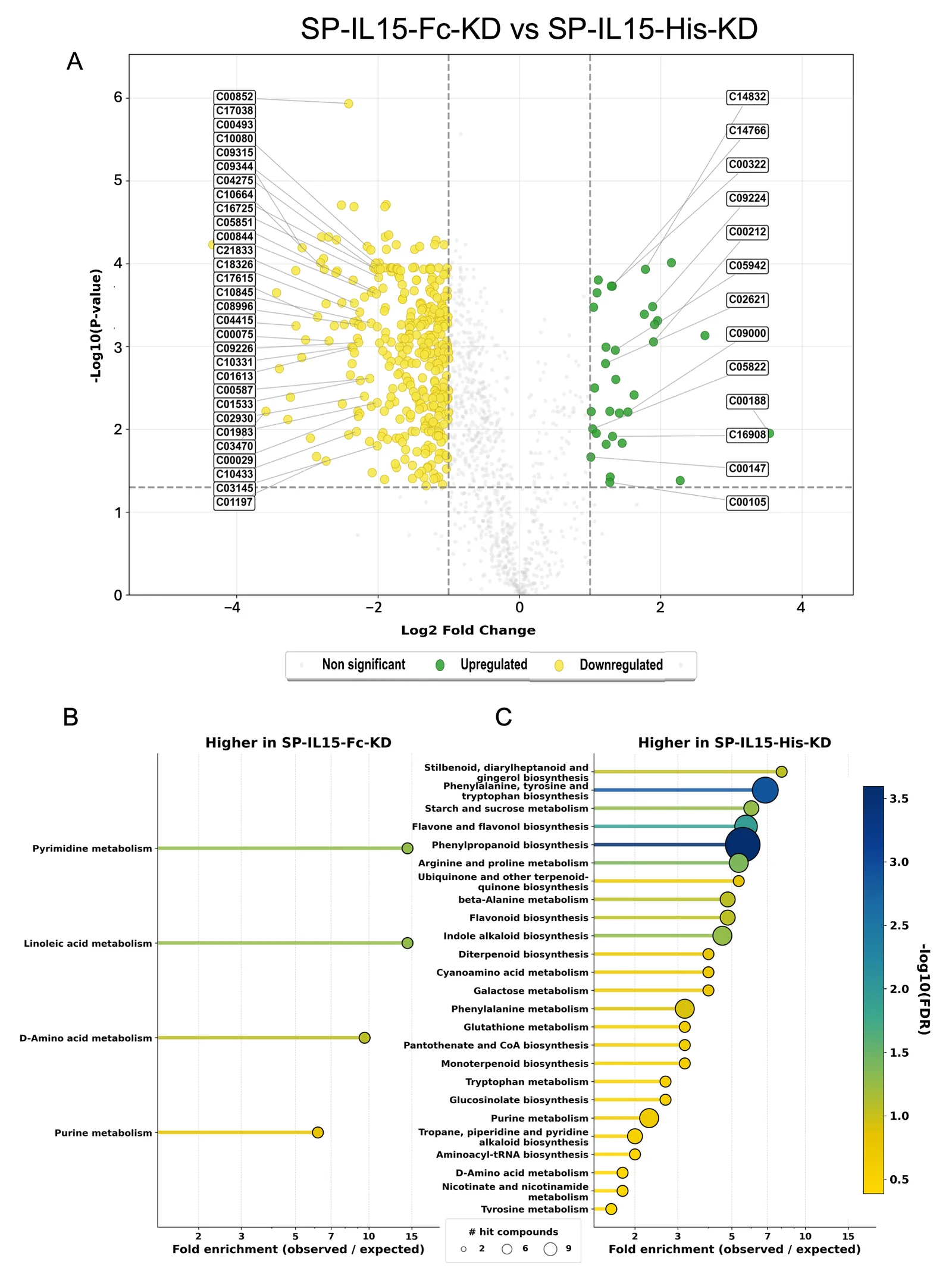
Differential metabolite abundance and pathway enrichment for the SP-IL15-Fc-KD and SP-IL15-His-KD. (**A**) Volcano plot of differentially abundant metabolites between the SP-IL15-Fc-KD and SP-IL15-His-KD constructs. Green and yellow points mark metabolites significantly higher in the Fc-fused construct and in its His-tagged counterpart, respectively; the x-axis shows log_2_ fold change and the y-axis shows −log_10_ of the *p*-value; vertical dashed lines mark |log_2_ fold change| = 1 and the horizontal dashed line marks the Benjamini–Hochberg FDR threshold of 0.05, and only significant features carrying a KEGG compound identifier are labelled. (**B**,**C**) KEGG pathway enrichment of the significant metabolites from SP-IL15-Fc-KD versus SP-IL15-His-KD: pathways enriched among metabolites higher in SP-IL15-Fc-KD (**B**) and higher in SP-IL15-His-KD (**C**). The x-axis gives the fold enrichment of each pathway (observed over expected), bubble size gives the number of hit compounds, and colour encodes the Benjamini–Hochberg-corrected *p*-value as −log_10_(FDR); pathways were considered significantly enriched at FDR < 0.05.

The same pairwise analysis was applied to the metabolomic dataset (Figure 7 and Figure 8, Supplementary Table S7). In the IL15-Fc versus IL15-His comparison, 333 features reached significance, 204 of them higher in IL15-Fc and 129 higher in IL15-His. Of these, 159 carried a KEGG compound identifier and were used for the pathway enrichment analysis. Enrichment significance was assessed after Benjamini–Hochberg correction, pooled across both comparisons and both directions, as for the proteins.

Phenylpropanoid-derived compounds were among the most strongly increased metabolites in IL15-Fc. Naringin (C09789), fraxin (C09266), coumarin (C05851), coumaroyl putrescine (C18326), the flavone apiin (C04858), kaempferol 3-O-glucoside (C12249), 1-O-sinapoyl-glucose (C01175), spermidine (C00315) and trans-ferulic acid (C01494) were all higher in IL15-Fc (Figure 7A, Table 2). These compounds map to phenylpropanoid biosynthesis, flavone and flavonol biosynthesis, flavonoid biosynthesis, and arginine and proline metabolism. At the pathway level, however, only monoterpenoid biosynthesis reached significance among the metabolites higher in IL15-Fc after correction, and the phenylpropanoid and flavonoid pathways did not (Figure 7B, Supplementary Table S9). The phenylpropanoid shift is supported by individual compounds but not by pathway enrichment.

The two members of the glutathione couple fell on opposite sides of the comparison. Oxidized glutathione (C00127) was higher in IL15-Fc, while reduced glutathione (C00051) was higher in IL15-His (Figure 7A, Table 2). These changes are consistent with an altered glutathione pool. Both were annotated to glutathione metabolism, which appeared on the IL15-Fc side of the enrichment but did not reach significance after FDR correction (Figure 7B, Supplementary Table S9).

The compounds higher in IL15-His fell into two classes: the first was the set of oxidized C18 fatty acids: C16325, C16321, C01226, C04780, and the further oxidized species C14766, C14832, C14827, and C14833 (Table 2). The second was a set of small organic acids and related compounds: 2-oxoadipate (C00322), phenol (C00146), fumarate (C00122), maleate (C01384), citrate (C00158) and pyruvate (C00022), together with adenosine (C00212), inosine 5′-diphosphate (C00104) and adenine (C00147). The first group maps to alpha-linolenic and linoleic acid metabolism, and the second to alanine, aspartate and glutamate metabolism, butanoate metabolism and tyrosine metabolism, all five of which were significantly enriched among the metabolites higher in IL15-His (Figure 7C, Supplementary Table S9).

In the proteome, peroxidase (K00430) and superoxide dismutase (K04565) were among the most strongly increased proteins in IL15-Fc and were annotated to the phenylpropanoid biosynthesis and peroxisome pathways (Figure 5B). The phenylpropanoid-derived compounds were more abundant in IL15-Fc, although the pathway-level enrichment did not remain significant in the enrichment analysis. Peroxidase reduces hydrogen peroxide to water and takes electrons from a phenolic donor, and the donors available in leaf tissue are the hydroxycinnamic acids and flavonoids of the phenylpropanoid pathway, several of which also remove peroxide directly [32,51].

The second route to peroxide removal runs on thiols rather than phenolics. Peroxiredoxins reduce peroxide through a catalytic cysteine and are returned to the active form by thioredoxin or glutaredoxin, and glutaredoxin in turn takes its electrons from reduced glutathione, which is oxidised to oxidized glutathione and recycled by glutathione reductase [50,56]. The glutaredoxin-dependent peroxiredoxin (K11188) and peroxiredoxin Q were more abundant in IL15-His, so heavier use of this route in IL15-His would be expected to leave more oxidised glutathione on that side, but the glutathione pool showed the opposite pattern. An increased demand for glutathione that is specific to IL15-Fc could offer an explanation, because reduced glutathione is consumed when disulfide bonds are formed, whereas the His-tagged construct does not require disulfide bond formation [57,58]. Protein disulfide-isomerase (K09580) and peptidyl-prolyl cis-trans isomerase (K09579) were more abundant in IL15-Fc (Figure 5B), which is consistent with increased demand on ER protein-folding and disulfide-bond processing machinery. IL15-His may use the thiol peroxidase route without the additional folding demand, which would leave its peroxiredoxins more abundant and its glutathione pool more reduced.

The organic acids follow the proteome in the same way. Ribose-5-phosphate isomerase (K01807), fructose-bisphosphate aldolase (K01623), enolase (K01689) and the oxygen-evolving proteins of photosystem II (K02716, K08901) were higher in IL15-His, and carbon fixation, central sugar metabolism and the glyoxylate and dicarboxylate pathway were enriched as well (Figure 5C). Pyruvate, fumarate and citrate are intermediates of those same pathways and were also higher in IL15-His, so primary carbon metabolism was enriched in IL15-His in both datasets. The oxidized C18 fatty acids are the one group with no counterpart in the proteome, because no enzyme of the oxylipin branch was detected in the proteomic dataset. Polyunsaturated C18 fatty acids in membranes are nevertheless oxidized directly by reactive oxygen species and yield hydroxy and keto derivatives of linoleic and α-linolenic acid [59]. The amount of oxidized fatty acid may reflect the extent of membrane oxidation.

To test whether this response depended on the subcellular localization, the same analysis was applied to SP-IL15-Fc-KD versus SP-IL15-His-KD (Figure 8A–C, Table 2). In total, 374 features reached significance, and the distribution was strongly skewed toward the His-tagged construct: 32 features were higher in SP-IL15-Fc-KD and 342 were higher in SP-IL15-His-KD. Of these, 173 carried a KEGG identifier and were used for pathway enrichment.

The compounds higher in SP-IL15-His-KD were again dominated by the phenylpropanoid branch. The products included coumaroyl hexoside (C04415), 1,2-di-O-sinapoyl-glucose (C04275), caffeic acid (C01197), chlorogenic acid (C00852), p-coumaroyl quinic acid (C12208), trans-ferulic acid (C01494) and trans-cinnamic acid (C00423). The precursors of the pathways, including shikimic acid (C00493), 3-hydroxybenzoic acid (C00587), quinic acid (C00296), tryptophan (C00078), phenylalanine (C00079) and phenylpyruvic acid (C00166) (Figure 8A, Table 2), shifted with them. These compounds map to phenylpropanoid biosynthesis, phenylalanine, tyrosine and tryptophan biosynthesis, flavone and flavonol biosynthesis, and arginine and proline metabolism, all of which were significantly enriched among the metabolites higher in SP-IL15-His-KD (Figure 8C, Supplementary Table S9).

Far fewer compounds were higher in SP-IL15-Fc-KD. They included L-allo-threonine (C00188), adenosine (C00212), the oxidized C18 fatty acids C14832 and C14766, pyrrole-2-carboxylic acid (C05942), 2-oxoadipate (C00322), uridine 5′-monophosphate (C00105), cytidine 3′-monophosphate (C05822) and adenine (C00147) (Figure 8A, Table 2). These metabolites map to pyrimidine metabolism and linoleic acid metabolism, neither of which reached significance (Figure 8B, Supplementary Table S9).

The proteome for this pair shows the same distribution. Peroxidase and superoxide dismutase changed sides in the presence of the SP/KD targeting cassette and were higher in the His-tagged construct (Figure 6A), and the phenolic compounds that supply them changed sides with them. The thiol antioxidants and chaperones that increased in SP-IL15-Fc-KD act on protein cysteines and on unfolded proteins rather than on small molecules, so few metabolite changes would be expected to accompany them, and few were found. Neither member of the glutathione couple reached significance in this comparison, so the change in glutathione pool is specific to the constructs without the SP/KD targeting cassette.

The metabolome is consistent with the direction the proteome reported in both comparisons. Where the proteome placed an enzyme on one side, the metabolome placed its substrates or products on the same side, both in the pair without the SP/KD targeting cassette and, after the reversal, in the pair carrying the signal peptide and KD motif. The metabolomic data also cover what the proteomic data do not: the oxidized state of the glutathione pool in the Fc construct without the SP/KD targeting cassette, which links the redox balance of the host to the folding demand of the fusion partner, the accumulation of oxidized C18 fatty acids in the His-tagged constructs, and the shift in the phenylpropanoid branch at its precursors as well as at its end products. The fusion partner and the targeting strategy are therefore associated with the host response together rather than independently. Because every group was agroinfiltrated, the differences reported here are differences between constructs, but each response still combines agroinfiltration with recombinant protein expression, and the two cannot be separated within this dataset. The four groups were designed as a 2 × 2 comparison of construct features against a shared agroinfiltration background, and the response to agroinfiltration itself was quantified separately in our previous study, which used the same expression system, the same host and the same analytical platform and included both mock-infiltrated and non-infiltrated controls [21]. The consequence for the present study is that every difference reported here is a difference between constructs, and the component of the host response that is caused by Agrobacterium alone cannot be estimated from these data. Subcellular localization was inferred from the presence or absence of the signal peptide and KD motif and was not verified. Visible tissue damage also differed between the constructs, and it was more pronounced in the pair carrying the signal peptide and KD motif. The extent of this damage was not measured, so the comparison between the two pairs may be influenced by the degree of cell injury as well as by the targeting signal. A difference in cell death is unlikely to account for the responses reported here, because dying tissue produces a graded response in which greater damage moves the same markers further in the same direction. The two comparisons behave in the opposite way. The proteomic and metabolomic profiles therefore describe the state of the tissue at the single time point. Time-resolved sampling, direct measurement of oxidative and ER stress, transcript-level confirmation and imaging-based confirmation of localization would refine the picture further.

## 4. Conclusions

We compared four IL-15 expression constructs in *N. benthamiana* that differed in fusion partner (His or IgG1 Fc) and in the presence of an N-terminal signal peptide together with a C-terminal KD motif. At 4 dpi, IL-15 was detected by Western blot following affinity purification only for the Fc-fused constructs. However, an IL-15-specific peptide (DVQLLENWVNVISDLKK) was detected by MS1 in all four constructs, indicating that the protein was present in the His-tagged constructs but likely accumulated below the detection limit of the Western blot.

Proteomic and metabolomic analyses further showed that the fusion partner and the SP/KD targeting cassette influenced the host response, with significant interaction effects indicating that their effects were not independent. In the absence of the SP/KD targeting cassette, the Fc partner was associated with higher abundance of antioxidant- and protein-folding-related proteins and phenylpropanoid-derived metabolites, whereas the His-tagged construct was associated with higher abundance of proteins involved in primary carbon metabolism and their related organic-acid intermediates. When the signal peptide and KD motif were present, these patterns were altered and, in several cases, reversed. The Fc-fused construct containing the SP/KD targeting cassette additionally showed a chaperone-associated proteomic response. Overall, the metabolomic patterns were broadly consistent with the proteomic changes, supporting a coordinated alteration of host metabolism and protein-associated responses.

These findings demonstrate that recombinant IL-15 construct design can influence the physiological state of the *N. benthamiana* host even when differences in protein accumulation are not readily apparent from Western blot analysis. However, the present conclusions are restricted to the four constructs examined at 4 dpi. Direct measurements of subcellular localization, ER and oxidative stress, recombinant protein abundance, and additional time points would be required to establish the underlying mechanisms and their temporal dynamics. From a plant molecular farming perspective, these results highlight that fusion partner and intracellular targeting should be evaluated not only for recombinant protein accumulation but also for their effects on host proteome and metabolism.

## Figures and Tables

**Table 1:** Key differentially abundant proteins.

KEGG Identifier	Protein Name	Function	log_2_FC IL15-Fc vs. IL15-His	Log_2_FC SP-IL15-Fc-KD vs. SP-IL15-His-KD
K00430	Peroxidase	Antioxidant system	+2.42	−1.75
K04565	Superoxide dismutase [Cu-Zn]	Antioxidant system	+2.62	−1.99
K11188	Glutaredoxin-dependent peroxiredoxin	Redoxins	−0.71	n.s.
N/A	Peroxiredoxin Q, chloroplastic	Redoxins	−3.02	+2.11
K03564	Thioredoxin-dependent peroxiredoxin (fragment)	Redoxins	n.s.	+0.39
K03386	Thioredoxin-dependent peroxiredoxin	Redoxins	n.s.	+2.40
K03676	Glutaredoxin	Redoxins	n.s.	+1.20
K09580	Protein disulfide-isomerase	Protein processing in ER	+1.31	n.s.
K09579	Peptidyl-prolyl cis-trans isomerase	Protein processing in ER	+2.47	+0.36
K04077	Chaperonin 60 subunit beta 2, chloroplastic	Chaperonin family (Cpn60)	n.s.	+0.76
K09490	Luminal-binding protein 5	Protein processing in ER	n.s.	+0.66
K03283	Heat shock cognate 70 kDa protein 2-like	Protein processing in ER	n.s.	+1.76
K01601	Ribulose bisphosphate carboxylase large chain	Calvin cycle	+2.56	−2.00
K01807	Ribose-5-phosphate isomerase	Pentose phosphate pathway	−1.53	+0.56
K01623	Fructose-bisphosphate aldolase	Glycolysis/Calvin cycle	−0.96	n.s.
K01689	Enolase (phosphopyruvate hydratase)	Glycolysis	−0.65	+0.48
K02716	Oxygen-evolving enhancer protein 1, chloroplastic-like	Photosynthesis	−1.20	+0.52
K08901	16 kDa subunit of oxygen-evolving system of photosystem II	Photosynthesis	−0.90	n.s.
N/A	23 kDa subunit of oxygen-evolving system of photosystem II	Photosynthesis	n.s.	+0.87

In each comparison, green cells show higher levels in the Fc-fused construct (positive log₂FC) and red cells show higher levels in the His-tagged construct (negative log₂FC).

**Table 2:** Key differentially abundant metabolites.

KEGG Identifier	Metabolite Name	Class	log_2_FC IL15-Fc vs. IL15-His	log_2_FC SP-IL15-Fc-KD vs. SP-IL15-His-KD
C01494	trans-Ferulic acid	Phenylpropanoids	+1.03	−1.19
C01175	1-O-Sinapoyl-glucose	Phenylpropanoids	+1.23	−1.01
C00852	Chlorogenic acid	Phenylpropanoids	n.s.	−2.42
C09789	Naringin	Flavonoids	+2.94	−1.07
C04858	Apiin	Flavonoids	+1.77	−1.67
C12249	Kaempferol 3-O-glucoside	Flavonoids	+1.74	n.s.
C09266	Fraxin	Coumarins	+2.49	−1.24
C05851	Coumarin	Coumarins	+2.23	−2.07
C18326	Coumaroyl putrescine	Phenolamides/polyamines	+2.02	−2.26
C00315	Spermidine	Phenolamides/polyamines	+1.21	−1.07
C00493	Shikimic acid	Shikimate/aromatic amino acids	n.s.	−2.15
C00079	Phenylalanine	Shikimate/aromatic amino acids	n.s.	−1.47
C00078	Tryptophan	Shikimate/aromatic amino acids	+1.23	−1.61
C00127	Oxidized glutathione	Glutathione metabolism	+1.23	n.s.
C00051	Reduced glutathione	Glutathione metabolism	−3.26 to −2.38	n.s.
C16325	FA 18:4 + 1O	Oxidised C18 fatty acids	−1.63 to −1.37	−1.28
C14766	FA 18:4 + 2O	Oxidised C18 fatty acids	−2.19	+1.32
C14832	FA 18:3 + 2O	Oxidised C18 fatty acids	−2.04 to −1.36	+1.78
C00158	Citrate	TCA cycle/glycolysis	−1.22	n.s.
C00122	Fumarate	TCA cycle/glycolysis	−1.36	n.s.
C00022	Pyruvate	TCA cycle/glycolysis	−1.02	n.s.
C00322	2-Oxoadipate	Organic acids	−4.10	+1.30
C05942	Pyrrole-2-carboxylic acid	Organic acids	−1.35	+1.36
C00146	Phenol	Tyrosine metabolism	−2.30	n.s.
C00212	Adenosine	Purine metabolism	−1.47	+1.90
C00147	Adenine	Purine metabolism	−1.04	+1.01
C00105	Uridine 5′-monophosphate	Pyrimidine metabolism	n.s.	+1.28
C05822	Cytidine 3′-monophosphate	Pyrimidine metabolism	n.s.	+1.04
C00188	L-allo-Threonine	Amino acids	+1.19 to +2.01	+3.54

In each comparison, green cells show higher levels in the Fc-fused construct (positive log₂FC) and red cells show higher levels in the His-tagged construct (negative log₂FC).

## Data Availability

The proteomic and metabolomic datasets generated and analyzed during the current study are available through the accession numbers JPST004762 and ST005023.

## Supplementary Materials

The following supporting information can be downloaded at: https://www.mdpi.com/article/10.3390/plants15182844/s1.
