# Supplementary material for: Fusion Partner and ER-Retention Signal Are Associated with Distinct Host Proteomic and Metabolic Responses During Interleukin-15 Production in Nicotiana benthamiana
Source: Plants (Basel). 2026 Sep 17;15(18):2844. doi: 10.3390/plants15182844 (PMC13610313; doi:10.3390/plants15182844)
Supplement: Supplementary file 1 [file plants-15-02844-s001.zip › SUPPLEMENTARY FIGURES.pdf]

SUPPLEMENTARY FIGURES

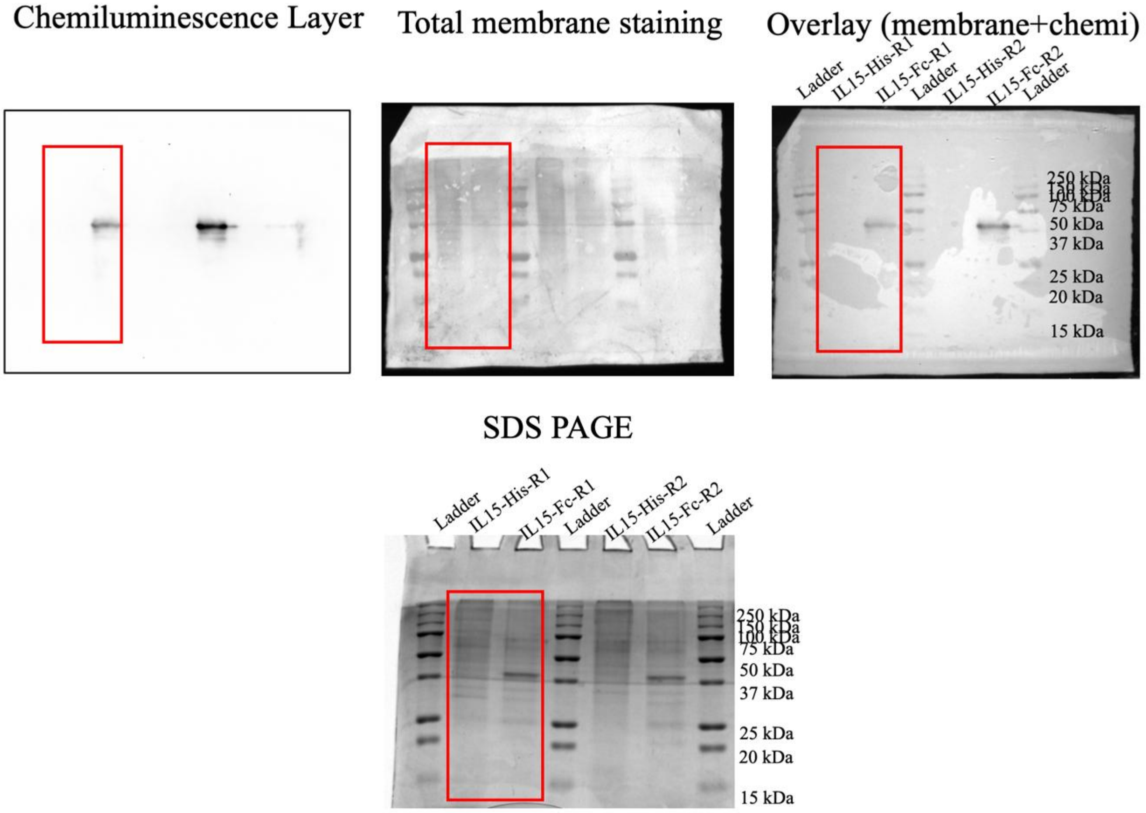

Supplementary Figure S1. Raw Western blot and SDS-PAGE images for IL15-His and IL15-Fc expression analysis. Chemiluminescence layer showing anti-IL15 detection of IL15-His and IL15-Fc with replicates. (Top left). Comassie-stained membrane as total protein stainning (Top center) Coomassie-stained SDS-PAGE gel of the same samples with molecular weight ladder (15–250 kDa). (Bottom panel) Overlay of membrane and chemiluminescence layers with molecular weight markers. Red boxes indicate the regions cropped for the corresponding main-text figure.

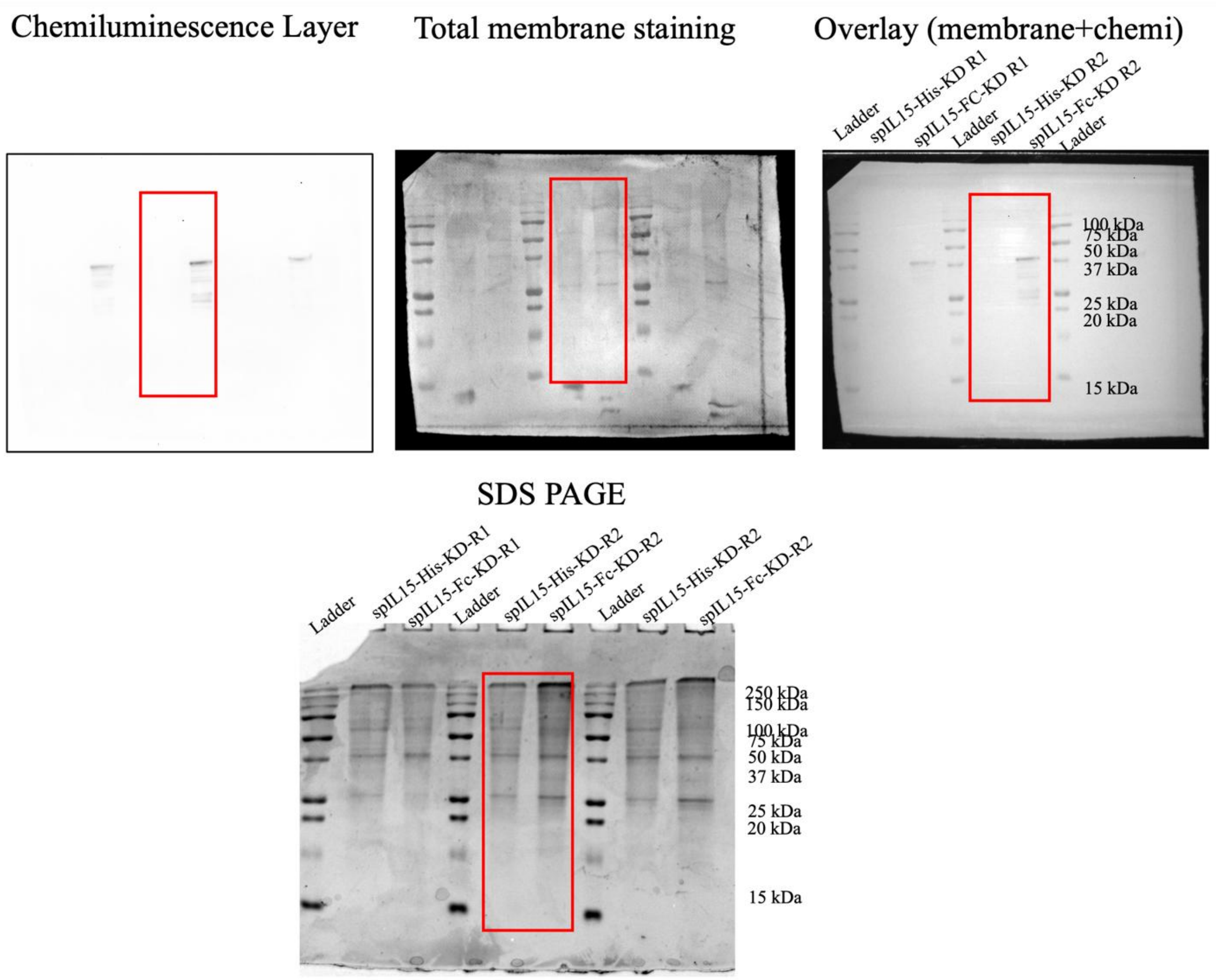

Supplementary Figure S2. Raw Western blot and SDS-PAGE images for SP-IL15-His-KD and SP-IL15-Fc-KD expression analysis. Chemiluminescence layer showing anti-IL15 detection of IL15-His and IL15-Fc with replicates. (Top left). Comassie-stained membrane as total protein stainning (Top center) Coomassie-stained SDS-PAGE gel of the same samples with molecular weight ladder (15–250 kDa). (Bottom panel) Overlay of membrane and chemiluminescence layers with molecular weight markers. Red boxes indicate the regions cropped for the corresponding main-text figure.

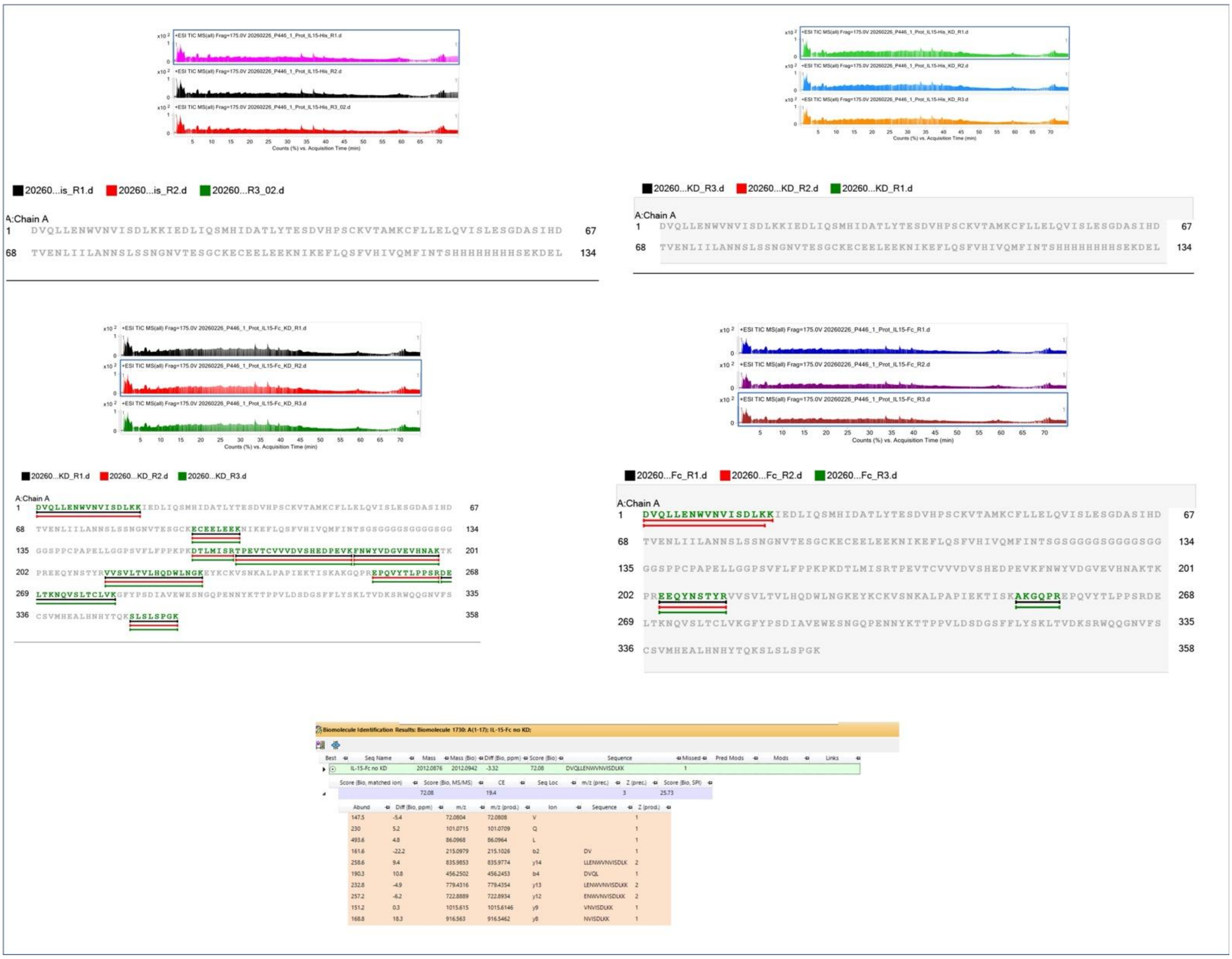

Supplementary Figure S3. LC-MS/MS peptide mapping of the four IL-15 constructs in crude leaf extracts. Tryptic digests of unpurified leaf tissue were analyzed by LC-MS/MS and searched against the corresponding construct sequence in Agilent MassHunter BioConfirm. Each quadrant shows one construct: IL15-His (top left), SP-IL15-His-KD (top right), SP-IL15-Fc-KD (bottom left) and IL15-Fc (bottom right). Within each quadrant, the upper trace set shows the total ion chromatograms of three biological replicates. and the lower panel shows the sequence coverage map for the same three replicates. Peptides identified by MS/MS are shown in bold green with a horizontal bar beneath, one bar per replicate, colored according to the key above each map. Grey residues were not covered. No peptides were identified in either His-tagged construct. Both Fc-fused constructs gave coverage across the IL-15 and Fc regions, including the IL-15-specific peptide DVQLLENWVNVISDLKK (residues 1–17). The lower panel shows the BioConfirm MS/MS identification of the peptide.

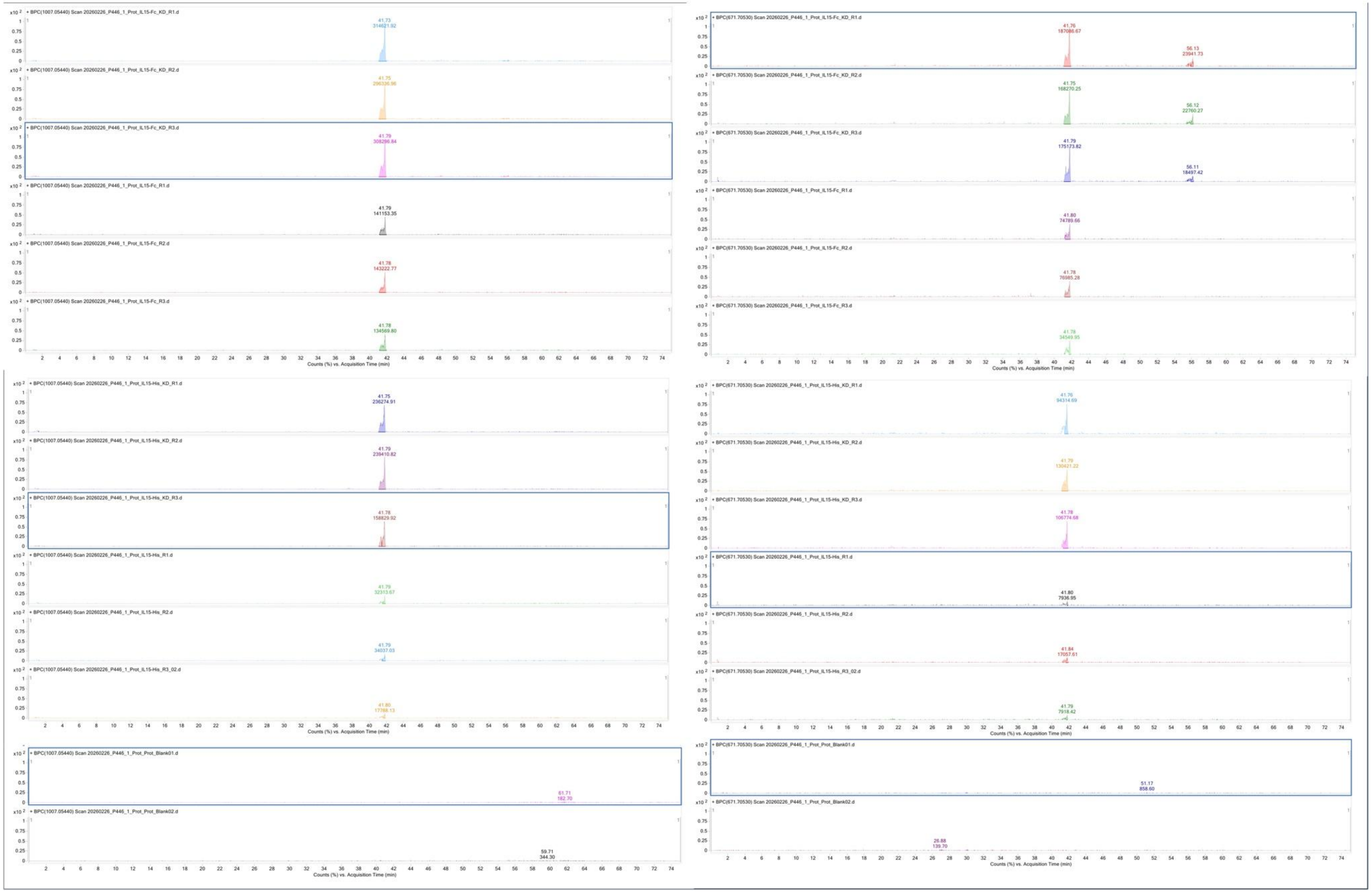

Supplementary Figure S4. Identification of the DVQLLENWVNVISDLKK peptide by exact mass. Extracted ion chromatograms of the IL-15-specific tryptic peptide DVQLLENWVNVISDLKK. Base peak chromatograms extracted for the doubly charged (m/z 1007.0544, left column) and triply charged (m/z 671.7053, right column) forms of DVQLLENWVNVISDLKK in the same LC-MS/MS runs shown in Figure S3. Traces are ordered from top to bottom: SP-IL15-Fc-KD (R1–R3), IL15-Fc (R1–R3), SP-IL15-His-KD (R1–R3), IL15-His (R1–R3) and two injection blanks. A peak at 41.7–41.8 min is present in all twelve tissue samples in both charge states and is absent from both blanks. Labels give the retention time and the apex abundance of each peak.
